# The anti‐human cytomegalovirus drug tricin inhibits cyclin‐dependent kinase 9

**DOI:** 10.1002/2211-5463.12398

**Published:** 2018-02-20

**Authors:** Hidetaka Sadanari, Kazuhiro J. Fujimoto, Yuto Sugihara, Tomoki Ishida, Masaya Takemoto, Tohru Daikoku, Tsugiya Murayama

**Affiliations:** ^1^ Center for Basic Education Faculty of Pharmaceutical Sciences Hokuriku University Kanazawa Japan; ^2^ Department of Microbiology and Immunology Faculty of Pharmaceutical Sciences Hokuriku University Kanazawa Japan

**Keywords:** anti‐cytomegalovirus agent, cyclin‐dependent kinase 9, cytomegalovirus replication, tricin

## Abstract

4′,5,7‐trihydroxy‐3′,5′‐dimethoxyflavone (tricin), derived from *Sasa albo‐marginata*, has been reported to suppress significantly human cytomegalovirus (HCMV) replication in human embryonic lung (HEL) fibroblast cells. However, the target protein of tricin remains unclear. This study focused on the anti‐HCMV activity of tricin in terms of its binding affinity to cyclin‐dependent kinase 9 (CDK9). A molecular docking study predicted that tricin binds well to the ATP‐binding site of CDK9. Experimental measurements then revealed that tricin inhibits the kinase activity of CDK9 and affects the phosphorylation of the carboxy‐terminal domain of RNA polymerase II. Based on these results, we conclude that CDK9 is one of the target proteins of tricin. We also found that tricin possesses anti‐HCMV activity with no cytotoxicity against HEL cells.

**Abbreviations**
CC_50_: 50% cytotoxic concentration
CDK9: cyclin‐dependent kinase 9
CTD: carboxy‐terminal domain
CycK: cyclin K
CycT1: cyclin T1
EC_50_: 50% effective concentration
F*l*ABCps: fitness learning‐based artificial bee colony with proximity stimuli
HCMV: human cytomegalovirus
HEL: human embryonic lung
hpi: hours postinfection
IC_50_: 50% inhibitory concentration
IE: immediate early
LDH: lactate dehydrogenase
RMSD: root‐mean‐square deviation
Ser2‐P: serine 2 phosphorylation
Ser5‐P: serine 5 phosphorylation
SI: selective index

Human cytomegalovirus (HCMV), also known as human herpesvirus 5, is a widespread viral pathogen that causes serious diseases in newborn infants and immunocompromised patients 1, 2, 3, 4. Highly potent drugs such as ganciclovir, valganciclovir, foscarnet, and cidofovir are now available for HCMV treatment. These compounds inhibit viral DNA synthesis by targeting the HCMV DNA polymerase (Enzyme Commission number: http://www.chem.qmul.ac.uk/iubmb/enzyme/EC2/7/7/7.html) 5. However, prolonged administration of anti‐HCMV agents induces antiviral drug resistance, leading to a recurrent problem in the treatment of immunocompromised patients. In addition, anti‐HCMV agents cause frequent adverse side effects such as bone marrow suppression 6, 7 and nephrotoxicity 6, 8, 9 because of their toxicities. To circumvent these problems, a novel type of anti‐HCMV drug is required.

4′,5,7‐trihydroxy‐3′,5′‐dimethoxyflavone (tricin; Fig. 1A), derived from *S. albo‐marginata*, has been shown to suppress significantly the replication of HCMV and influenza virus 10, 11, 12. Moreover, the compound has been shown to inhibit viral gene expression of immediate early (IE) 2 and UL54 (DNA polymerase) in a dose‐dependent manner 10. Furthermore, the action of tricin was found to affect the expression of chemokines 13, 14. These results indicate that tricin does not bind to HCMV DNA polymerase. However, the target protein of tricin remains unclear.

**Figure 1:**
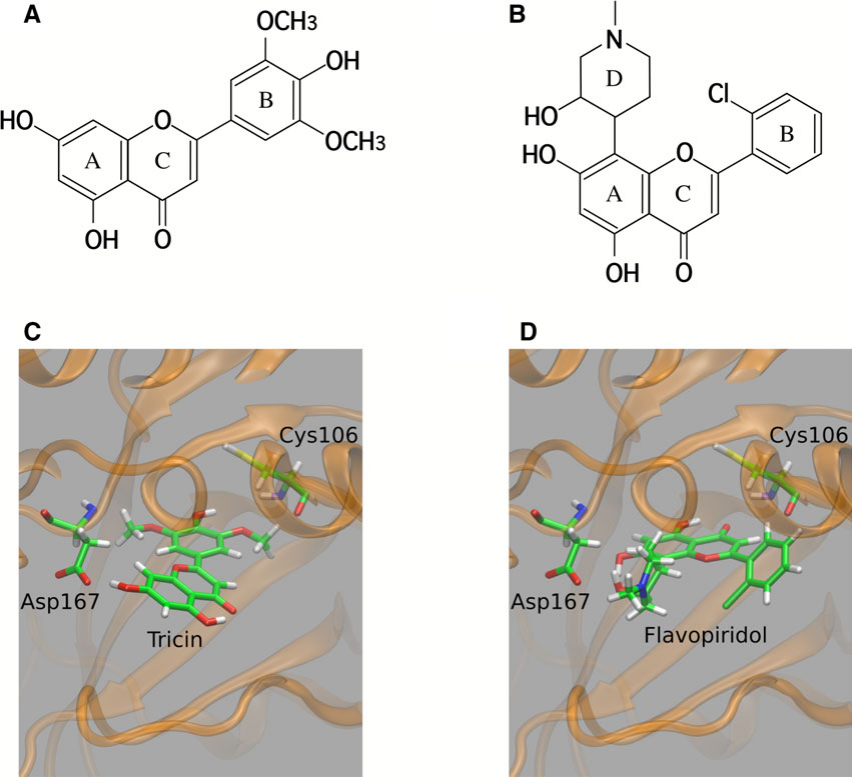
Chemical structures of (A) tricin and (B) flavopiridol. Docking poses of (C) tricin and (D) flavopiridol in the binding site of CDK9. In (A) and (B), the definitions of the rings, that is, A–C for tricin and A–D for flavopiridol, are also shown. In (C) and (D), the brown ribbon represents the backbone structure of CDK9. In (C), the OH groups in the A‐ and B‐ring of tricin are directed toward Asp167 and Cys106, respectively. In (D), the D‐ring of flavopiridol is located close to Asp167, and the carbonyl oxygen of the C‐ring is directed toward Cys106.

Cyclin‐dependent kinases (CDKs; http://www.chem.qmul.ac.uk/iubmb/enzyme/EC2/7/11/22.html) are known to be involved in viral replication 15, 16, 17, 18. It has been reported that cyclin‐dependent kinase 9 (CDK9)/cyclin T1 (CycT1) in mammalian cells initiates transcriptional elongation of genes by phosphorylating one serine residue, serine 2 (Ser2), within the carboxy‐terminal domain (CTD) of RNA polymerase II (RNA pol II) 19. In addition, CDK9 contributes to the phosphorylation of Ser5 near transcription start sites 20. Such phosphorylation activities of CDKs are inhibited by a synthetic flavonoid, flavopiridol (Fig. 1B) 21, 22, 23. X‐ray crystallographic analysis has revealed that flavopiridol binds to the ATP‐binding site of CDK9 24. As tricin is structurally similar to flavopiridol, these findings raise the possibility that tricin directly inhibits CDK9, thereby inhibiting viral RNA transcription.

In this study, we investigated whether tricin binds to CDK9 and suppresses viral RNA transcription. For this purpose, we first performed molecular docking simulations using a novel optimization algorithm called fitness learning‐based artificial bee colony with proximity stimuli (F*l*ABCps) 25, 26, 27, 28. The docking simulation successfully provided the binding conformation of tricin in the binding site of CDK9. Subsequently, we evaluated the *in vitro* kinase inhibitory activity of tricin against CDK9 and tested whether tricin affects *in vivo* the phosphorylation of CTD of RNA pol II. We also evaluated the cellular antiviral potency of tricin and its cytotoxicity, which were compared with those of flavopiridol.

## Materials and methods

### Molecular docking study

The structures of tricin and flavopiridol shown in Fig. 1A and B, respectively, were optimized with density‐functional theory at the B3LYP/6‐31G* level 29. Atomic charges of the ligands were derived from electrostatic potential fitting 30. All electronic structure calculations were performed with the Gaussian 09 program package 31. Atomic coordinates of human CDK9 were taken from the crystal structure (PDB ID: 3BLR 24). This crystal structure also contained flavopiridol. The molecular docking simulations with F*l*ABCps 27 were carried out for a cubic box (22.5 × 22.5 × 22.5 Å^3^) at the ATP‐binding site of CDK9, in which the AutoDock force field 32 was employed to calculate the scoring function. The maximum number of energy evaluations before the termination of F*l*ABCps was set to 2 500 000.

### Cell and virus

Human embryonic lung (HEL) fibroblast cells 33 were grown in Dulbecco's modified Eagle's medium (Nissui Pharmaceutical Inc., Tokyo, Japan) as described previously 10. HCMV strain Towne was used in all experiments. The history of this strain has been described elsewhere 34, 35. HCMV was propagated in HEL cells. Infectious virus production was titrated using a plaque assay as described previously 35.

### Compounds

Tricin was synthesized as described previously 10, 11, and flavopiridol was purchased from Cayman Chemical (Ann Arbor, MI, USA). Both compounds were dissolved at indicated concentrations as a stock solution in DMSO and stored at −80 °C until use. The final concentration of DMSO in cell culture was adjusted 0.1%.

### *In vitro* enzymatic kinase assay for CDK9

As CDK9 activation requires binding of CycT or cyclin K (CycK), we used a CDK9/CycK kinase assay in this study. Kinase activity and inhibition were measured by quantifying the amount of ADP converted from ATP during a kinase reaction by the ADP‐Glo™ kinase assay (Promega Japan Inc., Tokyo, Japan). Kinase reactions were performed in kinase reaction buffer [40 mm Tris (pH 7.5), 20 mm MgCl_2_, 0.1 mg·mL^−1^ BSA, and 50 μm dithiothreitol] supplemented with 10 μm ATP, CDK9/CycK kinase, and PDKtide synthetic peptide as substrates, according to the manufacturer's protocol. Briefly, kinase reactions were started by adding 2 μL of the ATP/substrate (final concentration of 0.2 μg·μL^−1^ PDKtide) to mixtures of 1 μL of 5× concentrated drug (diluted in final concentrations of 5% DMSO) and 2 μL of kinase solution (containing 30 ng CDK9/CycK), incubated for 2 h at room temperature (22–25 °C), and then stopped by adding 5 μL of ADP‐Glo reagent. After incubation of the reaction mixture at room temperature for 40 min, 10 μL of kinase detection reagent was added, and then the mixture was incubated for an additional 40 min. Subsequently, the luminescence intensity was measured by a luminometer (MiniLumat LB 9506, Berthold Technologies, Bad Wildbad, Germany). This assay is sensitive enough to detect very low amounts of ADP (20 nm) in a linear fashion. No‐enzyme and no‐inhibitor reactions represented background luminescence (0% activity) and noninhibited kinase activity (100% activity), respectively. The percent kinase activity was calculated by (a) subtracting the averaged value of no‐enzyme reaction luminescence from that of the kinase‐containing reaction with or without inhibitor and (b) converting these net luminescence values to percent activity based on no‐inhibitor reactions, which represented 100% kinase activity.

### Western blot analysis

Western blot analysis was performed as described previously 11. Briefly, cells were lysed in SDS sample buffer [62.5 mm Tris/HCl (pH 6.8), 2% SDS, 10% glycerol, 5% 2‐mercaptoethanol] and cell lysates were subjected to electrophoresis on 5–15% SDS/PAGE (Bullet PAGE One Precast gel; Nacalai Tesque Inc., Kyoto, Japan) and transferred to polyvinylidene difluoride membranes (GE Healthcare, Little Chalfont, UK (formerly Amersham Bioscience, Little Chalfont, UK)). The blots were blocked in TBS‐T [20 mm Tris/HCl (pH 7.5), 150 mm NaCl, 0.1% Tween 20] with 5% skim milk for 1 h and reacted with primary antibody diluted in TBS‐T plus 5% skim milk overnight at 4 °C. After washing with TBS‐T, the blots were incubated with horseradish peroxidase‐conjugated anti‐IgG (Cell Signaling Technology Japan Inc., Tokyo, Japan) in TBS‐T plus 5% skim milk for 1 h, washed in TBS‐T, and then developed by enhanced chemiluminescence according to the manufacturer's protocol (GE Healthcare). For a loading control, after detection of the protein of interest, the membrane was stripped and reprobed with anti‐β‐actin antibody conjugated with horseradish peroxidase (Abcam, Cambridge, UK) according to the Amersham ECL protocol. Anti‐phospho‐Ser2 RNA pol II and anti‐phospho‐Ser5 RNA pol II antibodies, which specifically recognize the Ser2‐ and Ser5‐phosphorylated forms within the CTD of RNA pol II, respectively, were purchased from Bethyl Laboratories (Montgomery, TX, USA).

### Plaque reduction assay

HEL cells were grown in 24‐well plates to more than 90% confluence and infected with HCMV at around 100 PFU per well. Following 90‐min adsorption, the medium was aspirated from the wells, and fresh medium containing selected drug dilutions of tricin and flavopiridol and 0.4% of agarose was added into triplicate wells. After incubation at 37 °C for 6–8 days, the cell monolayer was fixed with 10% formalin and then stained with 0.05% crystal violet. Plaques were counted microscopically under low power. Drug effects were calculated as the percent reduction in the number of plaques in the presence of each drug concentration to the number of plaques observed in the absence of drug.

### Cell toxicity assays

To measure cytotoxic effects of tricin and flavopiridol, the CytoTox 96 assay kit (Promega Japan Inc.) was used according to the manufacturer's instructions. The CytoTox 96 assay kit quantitatively measures a cytosolic enzyme, lactate dehydrogenase (LDH), which is released upon cell lysis. A total of 10 000 cells were plated in a 96‐well tissue culture plate in triplicate, and serum‐free medium containing various concentrations of the drug was present before the cytotoxicity assays. The released LDH is able to convert the substrate tetrazolium salt into a red formazan product, which can be measured at 492 nm in a 96‐well tissue culture plate. To determine the percent cytotoxicity, the amount of LDH released by cells after drug treatment was compared and normalized with the amount of LDH released after complete cell lysis.

## Results

### Tricin is predicted to strongly bind to CDK9

We first performed redocking calculations 1000 times with the crystal structure of CDK9 and flavopiridol (PDB ID: 3BLR). As a result, the lowest energy pose of flavopiridol successfully reproduced the crystal structure with a root‐mean‐square deviation (RMSD) of 0.53 Å. The docking poses with the RMSD < 1.0 Å were obtained 848 times out of 1000 runs. These results showed that the F*l*ABCps method is applicable to this system.

We next investigated whether tricin binds to CDK9. Molecular docking simulations with F*l*ABCps were performed 1000 times. Figure 1C shows the docking pose of tricin with the lowest energy in the binding site of CDK9. The A‐ring of the flavone skeleton is located close to Asp167, and the OH‐group in the B‐ring is directed toward Cys106. The docking poses of tricin with an RMSD < 1.0 Å from the lowest energy pose were obtained 485 times out of 1000 runs. Table 1 summarizes the predicted binding energies of the best scoring poses. The binding energy between tricin and CDK9 was calculated to be −5.77 kcal·mol^−1^. We also took the average of the 1000 binding energies, which yielded a result of −5.56 kcal·mol^−1^. From these results, tricin was predicted to bind well to CDK9.

**Table 1:** CDK9‐ligand binding energies (kcal·mol^−1^)

	Lowest value^a^	Mean value^b^
Tricin	−5.77	−5.56
Flavopiridol	−6.65	−6.56

a The lowest value of the 1000 runs.

b Mean value calculated with the 1000 binding energies.

The results of flavopiridol were also compared with those of tricin. Figure 1D illustrates the lowest energy pose of flavopiridol obtained with F*l*ABCps. As shown, the direction of flavopiridol is different from that of tricin. The D‐ring of flavopiridol is located close to Asp167, while the carbonyl oxygen of the C‐ring is directed toward Cys106. The binding energy of the best scoring pose and the mean value were calculated to be −6.65 and −6.56 kcal·mol^−1^, respectively. These results showed lower binding energies for flavopiridol than for tricin. Thus, flavopiridol was predicted to bind to CDK9 stronger than tricin.

### Tricin inhibits the kinase activity of the CDK9/CycK complex

**Figure 2:**
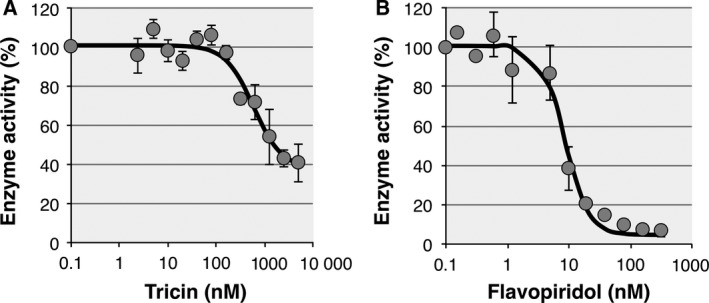
Effect of tricin on the kinase activity of the CDK9/CycK complex. Kinase assays were performed and quantitated as described in the Materials and methods. The lanes (left to right) are from reactions containing 2.44–5000 nm tricin (A) or 0.152–312.5 nm flavopiridol (B), both of which were prepared by making twofold serial dilutions. The percent kinase activity was calculated as described in the Materials and methods. The IC_50_ values were calculated by fitting the data to a logistic dose–response curve using curve‐fitting tools in NIH imagej software (National Institute of Health, Bethesda, MD, USA). Each inhibition curve was performed more than twice on different experiments.

Based on the computational results, we experimentally investigated the kinase inhibitory activity of tricin against CDK9. As shown in Fig. 2A, tricin inhibited the kinase activity of the CDK9/CycK complex in a dose‐dependent manner. Although the kinase activity at more than 10 μm tricin could not be assessed due to tricin precipitation, the 50% inhibitory concentration (IC_50_) of tricin was evaluated to be 1.38 ± 0.83 μm, which resulted in a higher value than those of other CDK9 inhibitors 36, 37. As mentioned above, flavopiridol is a widely known CDK inhibitor 21; thus, we also measured the kinase inhibitory activity of flavopiridol for comparison. As shown in Fig. 2B, the kinase activity of the CDK9/CycK complex was dramatically inhibited by flavopiridol. The IC_50_ of flavopiridol was calculated to be 8.20 ± 2.54 nm, similar to previously published values (4.59 nm 38 and 11 nm 39). From these results, we confirmed that tricin inhibits the kinase activity of CDK9 and that its inhibitory activity is weaker than that of flavopiridol, which correlates with the results of the docking simulations.

### Tricin inhibits the phosphorylation of Ser2 and Ser5 within the CTD of RNA pol II

**Figure 3:**
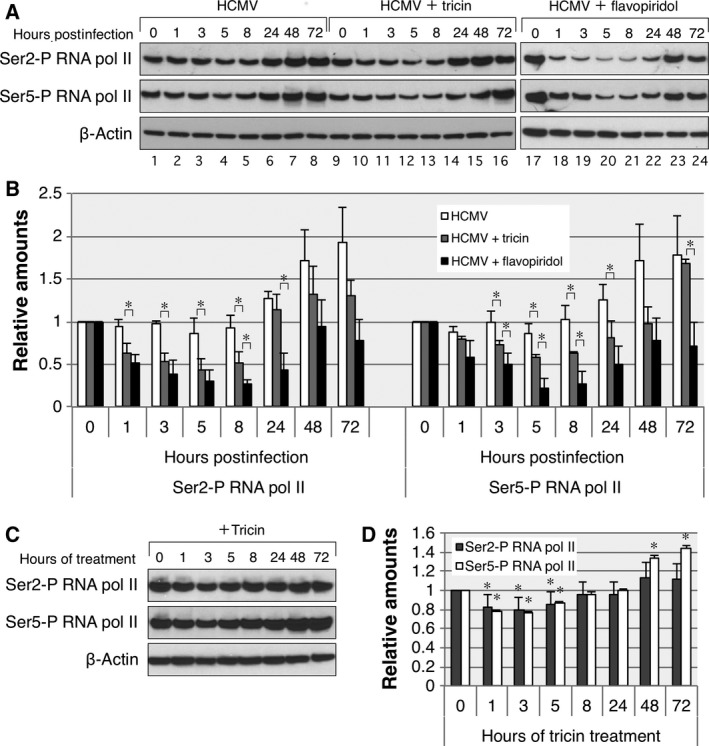
Effect of tricin on the levels of Ser2‐P and Ser5‐P in HEL cells. (A) HEL cells cultured to semiconfluence (more than 80%) were infected with HCMV at an MOI of 1 PFU per cell in the absence or presence of 10 μm tricin or 62.5 nm flavopiridol. At the indicated time points, cells were harvested in lysis buffer and the concentrations of Ser2‐P or Ser5‐P were determined by western blot analysis. (C) HEL cells cultured to semiconfluence (more than 80%) were treated with 10 μm tricin, and cells were harvested in lysis buffer at the indicated time points. (B and D) Band intensities were quantitated using NIH imagej software, and the levels were normalized to those of β‐actin (internal control). The results shown are the mean and SD of three different experiments. *P* values were calculated using Student's *t*‐test. *P <* *0.05, HCMV‐infected or HCMV‐infected and flavopiridol‐treated cells compared with HCMV‐infected and tricin‐treated cells (B); *P <* *0.05, tricin‐treated cells compared with uninfected and untreated cells (D).

We next investigated the effect of CDK9 inhibition by tricin on *in vitro* cell culture of HEL cells. One established cellular target of CDK9 is Ser2 and Ser5 within the CTD of RNA pol II. The inhibition of phosphorylation at these two sites has been reported to suppress the transcription of IE genes of HCMV 15. As shown in Fig. 3A, in HCMV‐infected HEL cells, the levels of Ser2 and Ser5 phosphorylation (Ser2‐P and Ser5‐P, respectively) remained relatively constant between 1 and 5 h postinfection (hpi). However, these levels dramatically increased to maxima until 48 hpi and remained at maxima at 72 hpi (Fig. 3A, upper and middle panels, lanes 1–8). Figure 3B shows the relative densitometric analyses of the blots, normalized to β‐actin levels. In uninfected HEL cells (Fig. 3B, white bars), the relative levels of Ser2‐P and Ser5‐P at 48 and 72 hpi were augmented 1.8‐fold or more than those at 0 hpi. In contrast, the levels of Ser2‐P and Ser5‐P gradually decreased until 5 hpi in HCMV‐infected cells treated with 10 μm tricin, then increased between 8 and 72 hpi (Fig. 3A, upper and middle panels, lanes 9–16). This time course study clearly demonstrated tricin treatment significantly reduces the relative levels of both Ser2‐P and Ser5‐P (Fig. 3B).

Next, the same analysis was performed with flavopiridol. As also shown in Fig. 3A, the levels of Ser2‐P and Ser5‐P quickly decreased at 1 hpi in HCMV‐infected cells treated with 62.5 nm flavopiridol. These levels remained relatively stable up to 8 hpi, and then gradually increased (Fig. 3A, upper and middle panels, lanes 19–24). Consequently, the temporal profile (time course) for flavopiridol treatment was almost the same as that for tricin treatment (Fig. 3B, black bars vs. gray bars).

We also performed the same measurements with mock‐infected HEL cells treated with tricin. As shown in Fig. 3C, the levels of Ser2‐P and Ser5‐P decreased slightly but significantly between 1‐ and 5‐h treatment and increased gradually until 72‐h treatment. The relative levels of Ser2‐P and Ser5‐P at 72‐h treatment increased to 1.1‐ and 1.4‐fold of those in untreated cells, respectively (Fig. 3D).

These results indicate that tricin inhibits the phosphorylation at Ser2 and Ser5 within the CTD of RNA pol II for up to 8 hpi and this inhibition continues until at least 72 hpi. As shown by black and gray bars in Fig. 3B, the inhibition levels of tricin on the phosphorylation seem to be weaker than those of flavopiridol. However, the temporal profile (time course) of the inhibition by tricin is found to be similar to that by flavopiridol (Fig. 3B). These results strongly suggest that the inhibition of phosphorylation at Ser2 and Ser5 by tricin is attributed to its inhibition of the CDK9 kinase activity, as reported for flavopiridol 23.

### Comparison of anti‐HCMV activity between tricin and flavopiridol

**Figure 4:**
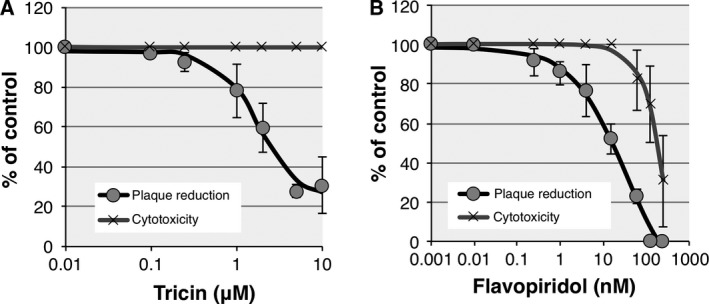
Comparison of anti‐HCMV activity and cytotoxicity between flavopiridol and tricin. HEL cells (1.5 × 10^5^ cells) plated in 24‐well plates were infected with HCMV and treated with various concentrations of flavopiridol or tricin during and after infection for 7 days. The antiviral activity was then determined in a PFU reduction assay. Cytotoxicity of the two compounds was determined by incubating HEL cells in 96‐well plates with various concentrations of flavopiridol or tricin for 7 days in triplicate, followed by measurement of LDH release as described in the Materials and methods.

To further investigate the anti‐HCMV effects of tricin and flavopiridol, the plaque reduction assay was performed. As shown by the black line in Fig. 4A, tricin had anti‐HCMV activity with a 50% effective concentration (EC_50_) of 2.09 ± 0.50 μm, which showed a similar value to the CDK9 kinase inhibitory activity (1.38 ± 0.83 μm). Conversely, the EC_50_ of flavopiridol was 15.8 ± 3.2 nm (Fig. 4B). This value was also similar to the CDK9 kinase inhibitory activity of flavopiridol (8.20 ± 2.54 nm). These results strongly suggest that the anti‐HCMV effects of tricin are also caused by the inhibition of the CDK9 kinase activity.

Although flavopiridol is well known as a potent CDK inhibitor 21, it is considered to be highly cytotoxic 22. For this reason, we next investigated the cytotoxic effect of tricin and flavopiridol in HEL cells. As shown by the gray line in Fig. 4A, tricin had no cytotoxic effect at all concentrations tested in this experiment. Conversely, flavopiridol was cytotoxic at concentrations approximately 10‐fold higher than the EC_50_ value obtained by the plaque reduction assay (Fig. 4B, gray line). Evaluation with the LDH leakage assay provided a 50% cytotoxic concentration (CC_50_) of 176 ± 29 nm for flavopiridol. Taking into account the EC_50_ value of flavopiridol (15.8 ± 3.2 nm), the selective index (SI = CC_50_/EC_50_) of flavopiridol was calculated to be 11.1, indicating low to moderate potential as an antiviral agent. From these results, we could confirm that flavopiridol possesses not only strong anti‐HCMV activity but also strong cytotoxicity. In contrast, no cytotoxic effect was observed for tricin in the present experiment; thus, tricin is considered the preferred candidate for anti‐HCMV drug development.

## Discussion

In this study, we investigated the anti‐HCMV effect of tricin in terms of its binding affinity to CDK9. Molecular docking study with the F*l*ABCps method predicted that tricin binds well to the binding site of CDK9. As X‐ray crystallographic analysis was not performed in this study, we cannot compare the predicted pose of tricin with the crystal structure. However, the predicted structure of tricin is considered to be reliable because F*l*ABCps successfully reproduced the correct binding pose of flavopiridol with an RMSD of 0.53 Å from the crystal structure. It should be noted that redocking with crystal structure is not a trivial problem for molecular docking study 40. A docking pose with an RMSD of < 2 Å is commonly regarded to indicate successful reproduction of the crystal ligand. We also calculated the binding energy of the crystallographic position of flavopiridol, which yielded a result of −6.13 kcal·mol^−1^. This value was larger by 0.52 kcal·mol^−1^ than the lowest binding energy obtained with F*l*ABCps (−6.65 kcal·mol^−1^). As mentioned above, the lowest energy pose of flavopiridol was almost the same as the crystal structure (RMSD = 0.53 Å); thus, these results may indicate that a slight difference in the ligand structure (e.g., positions of hydrogen atoms) strongly influences the interactions between CDK9 and flavopiridol. Molecular docking study also predicted that the binding affinity for tricin to CDK9 is weaker than that of flavopiridol to CDK9. Despite their similar structures, the binding poses of tricin and flavopiridol showed different directions, which resulted in different binding energy values.

The computational predictions were next verified experimentally by measuring the kinase inhibitory activity of tricin against CDK9, where flavopiridol was also evaluated for comparison. As predicted by the docking simulations, the experimental results clearly demonstrated that tricin inhibits the CDK9 kinase activity, while the IC_50_ value of tricin was approximately 170‐fold higher than that of flavopiridol. Subsequently, we investigated the influence of tricin on the phosphorylation at Ser2 and Ser5 within the CTD of RNA pol II. The experimental analysis revealed that tricin inhibits the phosphorylation of both Ser2 and Ser5, and its time course profile is similar to that of flavopiridol. Flavopiridol is known to reduce the phosphorylation activity of CDK9 at Ser2 and Ser5 within the CTD of RNA pol II. Therefore, the present results strongly suggested that the reduction in the phosphorylation at Ser2 and Ser5 by tricin is attributed to the inhibition of the CDK9 kinase activity.

We also evaluated the cellular anti‐HCMV potencies of tricin and flavopiridol. Tricin was shown to have anti‐HCMV activity, although its potency was weaker than that of flavopiridol. Moreover, the EC_50_ value of tricin on the anti‐HCMV activity was shown to be comparable to the IC_50_ value on the CDK9 kinase inhibitory activity. These results could be interpreted that the anti‐HCMV effects of tricin are caused by the inhibition of the CDK9 kinase activity. Based on these results, we concluded that CDK9 is one of the target proteins of tricin.

We finally measured the cytotoxicity of tricin and flavopiridol. Flavopiridol demonstrated strong cytotoxicity against HEL cells, while no cytotoxic effect was observed for tricin. Furthermore, the SI value of flavopiridol was estimated to be 11.1, which indicated that flavopiridol has low to moderate potential as an antiviral agent. Conversely, Sakai *et al*. 41 reported that the SI value of tricin was 1206. Taken together, these results suggest that tricin is the preferred candidate for anti‐HCMV drug development.

In this study, we focused on the CDK9 kinase inhibitory activity of tricin and concluded that CDK9 is one of the target proteins of tricin. On the other hand, we have also revealed that the action of tricin affects the expression of chemokines 13, 14. These results strongly suggest that the inhibition of HCMV replication by tricin cannot be explained by its effect on CDK9 alone. It is necessary to address whether the activity of other CDKs is inhibited by tricin and consequently which steps of HCMV replication are disrupted. Such studies will be performed in our future work.

## Author contributions

KJF and TM conceived and supervised the study; HS and TM designed experiments; KJF performed simulation studies; HS, YS, and TI performed experiments; HS, MT, and TD analyzed data; KJF and HS wrote the manuscript; TM, MT, and TD made manuscript revisions.
